# Inter-Specific Competition, but Not Different Soil Microbial Communities, Affects N Chemical Forms Uptake by Competing Graminoids of Upland Grasslands

**DOI:** 10.1371/journal.pone.0051193

**Published:** 2012-12-07

**Authors:** Eduardo Medina-Roldán, Richard D. Bardgett

**Affiliations:** Soil and Ecosystem Ecology Laboratory, Lancaster Environment Centre, Lancaster University, Lancaster, United Kingdom; University of Alberta, Canada

## Abstract

Evidence that plants differ in their ability to take up both organic (ON) and inorganic (IN) forms of nitrogen (N) has increased ecologists’ interest on resource-based plant competition. However, whether plant uptake of IN and ON responds to differences in soil microbial community composition and/or functioning has not yet been explored, despite soil microbes playing a key role in N cycling. Here, we report results from a competition experiment testing the hypothesis that soil microbial communities differing in metabolic activity as a result of long-term differences to grazing exposure could modify N uptake of *Eriophorum vaginatum* L. and *Nardus stricta* L. These graminoids co-occur on nutrient-poor, mountain grasslands where *E. vaginatum* decreases and *N. stricta* increases in response to long-term grazing. We inoculated sterilised soil with soil microbial communities from continuously grazed and ungrazed grasslands and planted soils with both *E. vaginatum* and *N. stricta*, and then tracked uptake of isotopically labelled NH_4_^+^ (IN) and glycine (ON) into plant tissues. The metabolically different microbial communities had no effect on N uptake by either of the graminoids, which might suggest functional equivalence of soil microbes in their impacts on plant N uptake. Consistent with its dominance in soils with greater concentrations of ON relative to IN in the soluble N pool, *Eriophorum vaginatum* took up more glycine than *N. stricta*. *Nardus stricta* reduced the glycine proportion taken up by *E. vaginatum*, thus increasing niche overlap in N usage between these species. Local abundances of these species in mountain grasslands are principally controlled by grazing and soil moisture, although our results suggest that changes in the relative availability of ON to IN can also play a role. Our results also suggest that coexistence of these species in mountain grasslands is likely based on non-equilibrium mechanisms such as disturbance and/or soil heterogeneity.

## Introduction

The ability of plants to directly take up organic nitrogen (ON) might constitute an important mechanism regulating plant species coexistence [1]–[4]. For instance, if plants species show differential N uptake for ON or inorganic N (IN), niche overlap and competition intensity for N could decrease [2]. Since the importance of ON in the ecosystem N pool increases as primary productivity decreases [5], [6], there might also be some degree of niche differentiation in N chemical use among plants with contrasting abundances along environmental gradients [7]. However, studies on plant N chemical form uptake have shown conflicting results, with plant species from different habitats displaying greater uptake for one N form [2], [8], or no differential uptake at all [3], [9], [10]. No differential uptake for N among plant species that differ in habitat might suggest weak niche differentiation, but other factors could also alter patterns of plant N uptake. For example, inter-specific competition [11]–[13] and soil microbial community composition and activity [14], [15] could alter N uptake among plant species. While the role of plant competition on N uptake patterns has recently been addressed [11], less is known about how soil microbes and changes in microbial communities affect plant uptake of different chemical forms of N [15], despite microbes being key agents in the N cycle.

A key factor that modifies soil microbial communities and nutrient cycling in grasslands is grazing by large herbivores. Moreover, grazing-induced changes in soils can then feedback to influence plant species performance and competition [16]–[20]. For instance, Medina-Roldán et al. [21] found that grazing-induced increases in soil microbial activity and soil N availability in temperate acid grasslands increased the competitive ability of *Nardus stricta* L. relative to *Eriophorum vaginatum* L. (nomenclature follows [22]), which might partly explain the dominance of the former species in grazed grassland. However, whether grazing impacts on soil mediate plant uptake of different chemical forms of N (ON and IN) remains unexplored.

Here, we report results from an experiment designed to test how grazing induced changes on soil microbial communities and soil properties affect N uptake patterns for ON (in the form of glycine) and IN (in the form of NH_4_^+^) in two dominant graminoids of temperate acidic grasslands. The two graminoids are *E. vaginatum*, which typically occurs at low abundance in grazed grassland, and *N. stricta*, a grazing-increaser. Additionally, we tested how glycine and NH_4_^+^ uptake by these two graminoids was affected by their competitive interactions. We tested three hypotheses. First, we hypothesized that the more metabolically-active soil microbial community of grazed grasslands [21], [23] would facilitate N uptake in both plant species, although the increase would be larger for *N. stricta* due to its greater competitive ability. Second, we hypothesized that *E. vaginatum* would take up more glycine than *N. stricta*, since the former is known to grow in soils with higher proportion of ON relative to IN [23]. Finally, we hypothesized that *N. stricta* would alter the N uptake patterns of *E. vaginatum* because of its higher competitive ability. These hypotheses were tested in a plant competition glasshouse experiment using soil inoculum from grazed and ungrazed acidic grasslands previously shown to differ in soil biological properties. Then, we used ^15^N labelled IN and dual-labelled ^15^N-^13^C amino acids to track the uptake these N compounds by the two graminoids.

## Materials and Methods

### Experimental Design

#### Soil substratum and inoculum preparation

**Figure 1:**
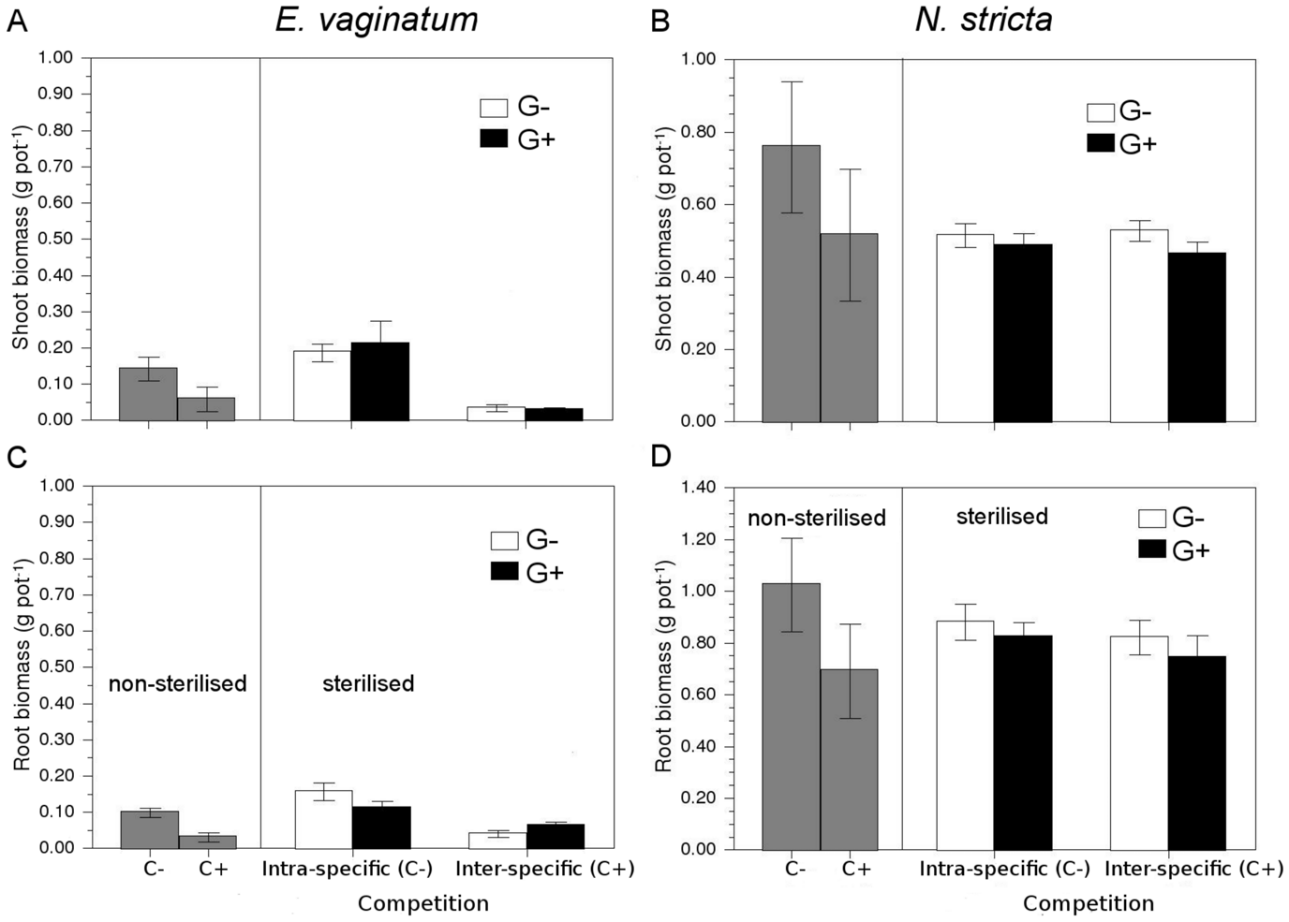
Plant-soil feedbacks effects on competition. Effect of microbial inoculum from a grazed *Nardus*-dominated acidic semi-natural grassland (G+) and a *Eriophorum*-dominated ungrazed grassland (G−), and inter-specific competition on: (a) *E. vaginatum* shoot biomass; (b) *N. stricta* shoot biomass; (c) *E. vaginatum* root biomass; and (d)*N. stricta* root biomass. Data show the effects of inter-specific competition when both plants were grown without (C−) or with (C+) inter-specific competition. Gray bars (NA) indicate the effect of competition on each species biomass component on non-inoculated soils. Values are means ±1 s.e.

Our study area is located in the Ingleborough National Nature Reserve, Yorkshire Dales, northern England (54.18° N, 2.36°E). On July 2010, soils were collected from a continuously-sheep grazed acidic grassland dominated by *N. stricta*, *Agrostis capillaris* L. and *Festuca spp,* and an adjacent area where grazing was excluded by fencing in 2000 (for more details about the areas see [23]). Exclusion of sheep grazing has led to dominance of the dwarf-shrub *Calluna vulgaris* (L.) Hull, and the graminoids *Deschampsia caespitosa* (L.) Beauv. and *E. vaginatum*, and it has reduced soil N availability, soil microbial activity, soil microbial biomass N, and the ratio of IN to ON in comparison with the adjacent continuously-grazed grassland [21], [23]. We collected soil from 5–7 sampling points on each of the grazed and ungrazed areas, and made a composite sample. A fraction of the composite sample was used as substrate and the other was used for the preparation of the soil inoculum (soil for inoculum was stored at 4°C until inoculum preparation on September 2010, see below). The fraction of soil to be used as substrate was mixed with sand in a 1∶5 ratio to have enough substrate for the experiment. After mixing, substrate was sterilised by autoclaving. To overcome side effects of autoclaving, substrate was air-dried before sterilisation [24]. Autoclaving did not affect significantly DOC and DON concentrations, microbial biomass C and N, or soil basal respiration in the substrate (Table 1). Additionally, autoclaving did not modify plant competition outcome in an independent competition assay (Fig. 1). We used 250 g of autoclaved substrate to fill up plastic pots (10 cm diameter, 9.5 cm height), which were set to 25% gravimetric soil moisture (65% of soil water holding capacity).

**Table 1:** Comparisons (means and s.e) of properties between non-sterilised or sterilised soil substratum used in the microcosms.

Soil variable	Non-Sterile	df	Sterile	|*t*|	*P*
Cmic	0.45 (0.45)	2.2	16.1 (16)	0.7	>0.50
Nmic	17 (1.9)	2.2	14 (8.2)	0.2	>0.80
DOC	350 (24)	2.2	587 (174)	1.4	>0.20
DON	4.5 (0.9)	2.2	15.0 (4.4)	2.3	>0.10
Bas. resp	2.5 (0.8)	2.2	2.9 (0.7)	0.3	>0.70

Cmic = carbon in microbial biomass (mg C kg soil^−1^), Nmic = nitrogen in microbial biomass (mg N kg soil^−1^), DOC = dissolved organic C (mg C kg soil^−1^), DON = dissolved organic nitrogen (mg N kg soil^−1^) and Bas. resp = soil basal respiration (µL CO2 g soil^−1^ h^−1^). |*t*| = absolute value for Welch Two Sample t-test and *P* = associated probability. n = 3. df = freedom degrees.

On September 2010, we inoculated the pots with soil microbial communities from the grazed and ungrazed areas. Soil inoculum was prepared by passing fresh soils through a 2 mm sieve and mixing them with a sterile weak Ringer solution (NaCl = 2.25, KCl = 0.105, CaCl_2_·6H_2_O = 0.12, NaHCO_3_ = 0.05, g per litre respectively) as described in Griffiths et al. [25] in a 0.5∶1 fresh soil to solution ratio (w/v). Microcosms were inoculated with 30 mL of inoculum assigned randomly from either grazed or ungrazed soil inocula. After inoculation, plants were immediately planted (see below).

#### Plant species establishment and competition treatments

Two-week old seedlings of *N. stricta,* derived from plants collected in our field sites and propagated in the glasshouse, were transferred into the microcosms immediately after soil inoculation early in September 2010. On the other hand, *E. vaginatum* seeds (also from plants collected in our field sites and propagated in the glasshouse) were planted 1 week after *N. stricta* in order to avoid large differences in plant size. Since competition between these graminoids occurs even at low densities [21], we used 2 plant individuals per microcosm in a substitution design. Thus, previously grazed- or ungrazed-inoculated microcosms were assigned randomly to the following plant competition treatments: 1) *N. stricta* intra-specific competition; 2) *E. vaginatum* intra-specific competition; and 3) inter-specific competition with one individual of each species per microcosm. Plants were allowed to grow for another 8 weeks and then we applied the ^15^N labelling treatments to track uptake of glycine and NH_4_^+^ (see below for treatments and number of replicates and [21] for glasshouse conditions).

#### ^15^N and ^13^C labelling, and microcosms’ harvest

Plant N uptake was assessed by tracking ^15^N and ^13^C labelled compounds according to the approach of Weigelt et al. [7]. In this method, plant IN and ON uptake is resolved by using solutions made of mixtures of N compounds where only one member in the mixture is either ^15^N labelled (IN) or dual ^13^C-^15^N labelled (ON). We used glycine (ON form) and NH_4_^+^ (IN form) which are dominant N forms in acid grasslands and moorlands [3], [26]. Discussions on limitations and strengths of the technique can be found elsewhere [3], [27]–[29]. Three N solutions (labelled NH_4_^+^  =  ^15^N-98%, dual labelled glycine = ^13^C 98%; ^15^N 98%, CK Gas Products Ltd.) containing both N compounds in equal N molarities per solution were used: (1) ^15^NH_4_^+^ + unlabelled glycine; (2) dual-labelled glycine+unlabelled NH_4_^+^; and (3) both glycine + NH_4_^+^ unlabelled as a negative control. Each solution was randomly assigned to microcosms where a combination of inoculum source and competition treatments had already been applied. Each inoculum source (grazed vs ungrazed)×competition (*N stricta* intra-specific vs *E. vaginatum* intra-specific vs inter-specific competition)×labelled N solutions (3 levels see above) combination had 4 replicates (2×3×3×4, n = 72). A solution with only distilled water was added to 4 additional microcosms (INAM) to determine isotopic natural abundances. Nitrogen solutions were applied by adding 10 µg N g dry soil^−1^ in 5-aliquots of 1 mL evenly distributed all through microcosms using a glass syringe with a 152 mm needle with sealed tip and 4 side ports. A short-term labelling period of 48 hrs was used in order to minimise ^15^N dilution [7], [30]. After 48 hrs, microcosms were harvested and shoot biomass for each species was oven-dried at 70°C for 48 hrs and weighed. Roots were separated from the soil, sorted by species in the case of the inter-specific competition treatment, rinsed in 0.5 M CaCl_2_, thoroughly washed under tap water to eliminate external isotopic traces, oven-dried and weighed as for shoot biomass.

### Laboratory Assays

Dried shoot and root biomass were ground in a ball-mill (biomass of both individuals was pooled for intra-specific competition treatments) and sent to the Stable Light Isotope Facility in the University of Bradford, United Kingdom, for analysis of elemental C and N, and isotopic mass ratio of ^13^C/^12^C and ^15^N/^14^N in a Thermo Finnigan Delta Plus XL continuous flow mass spectrometer equipped with a Flash EA 1112 elemental analyser. Following Näsholm et al. [31], we calculated isotopic enrichment (also called molar excess) in plant tissue as:
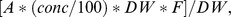



where conc = N or C content in plant tissue (%), DW is dry plant biomass, and F is the reciprocal of the molar mass of the isotopic species in question (either ^15^N or ^13^C). In the previous formula A is: 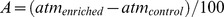, this is the difference in atm % between the enriched N solutions and the distilled water treatment [32].

Soil collected after harvest was stored at 4°C until laboratory analysis took place (within 3 weeks after harvesting). We determined soil microbial biomass C and N using the fumigation extraction technique [33]. Five g of fresh soil were extracted in 0.5 M K_2_SO_4_ by shaking the soil-extract for 30 min in an orbital shaker and filtering the soil extract in Whatman paper No. 1. Microbial biomass C is the difference in C concentrations between fumigated and non-fumigated extracts as measured in a Shimadzu 5000A TOC analyser (Shimadzu Inc., Japan) using an extraction efficiency of 0.45 [34]. Microbial biomass N was assayed by digesting the extracts with potassium persulfate [35]. Microbial biomass N is the difference in total N concentrations as measured with continuous-flow colorimetry in a Bran and Luebbe AutoAnalyzer 3 between fumigated and non-fumigated extracts using an extraction efficiency of 0.54 [36].

### Statistical Analysis

Effects of inoculum source, representing distinct soil microbial communities from grazed vs ungrazed grassland, and plant competition (intra-specific vs inter-specific) on shoot and root biomass of individual plants (averaged weight for the two individuals in intra-specific competition treatments) were tested using analysis of variance (ANOVA) models for each plant species separately. We used all microcosms for this analysis (including N solution mixtures plus INAM microcosms, around 51 observations per species), but without including N solutions as an experimental factor (no effect of N solutions on biomass was detected).

For molar excess values (which indicate N uptake), we analysed ^13^C and ^15^N separately. First, we tested effects of inoculum source, competition and plant species on ^13^C molar excess, albeit only for microcosms labelled with dual-labelled glycine (i.e., no ^13^C enrichment could be present in other N solutions). Inoculum source and competition were implemented as described above for biomass models. Additionally, we tested differences between our graminoids in ^13^C molar excess by including species nested within competition (since species are not crossed across intra- and inter-specific competition treatments) as a third factor in the model. For ^15^N molar excess, we tested effects of inoculum source, competition, and species nested within competition as described above. Additionally, we tested differences in ^15^N molar excess between glycine and NH_4_^+^ solutions (this difference is referred as to plant preference for glycine *vs* NH_4_^+^) by including ^15^N solution (glycine vs NH_4_^+^ source) in the models. As is customary in studies on plant uptake of different N forms [3], [7], [9], [31], we analysed shoot and root biomass data separately with both the ^13^C and ^15^N models. Finally for soil microbial biomass C and N, and its C: N ratio, we tested effects of inoculum source and plant species using ANOVA. The effect of plant species identify was implemented by using pot-type as a factor with 3 levels (only *E. vaginatum* or *N. stricta* presence, or both species presence) in the models.

All data were transformed for tests to satisfy normality criteria, but we use original values in the plots. We did not include the negative control (solution with both glycine and NH_4_^+^ unlabelled) in ^15^N and ^13^C models because of its high number of zeros. Because of this exclusion and analyses’ particularities (i.e., models for ^13^C have lower n’s), sample sizes used in models vary from the overall experimental sample size. We show this negative control in the plots to present a visual comparison. All analyses were performed with R for Linux [37].

## Results

### Plant Biomass

Inter-specific competition reduced *E. vaginatum* shoot biomass 85% (*F*_1,47_ = 50.0, *P*<0.001, Table 2) in comparison with *E. vaginatum* experiencing intra-specific competition only (Fig. 1a and 1c respectively). There was a significant inoculum source×competition interaction for *E. vaginatum* root biomass (*F*_1,43_ = 4.5, *P* = 0.04, Table 2) because inter-specific competition decreased root biomass more in the ungrazed (73% reduction) than in the grazed soil (45% reduction) (Fig. 1c). Unlike *E. vaginatum*, *N. stricta* shoot and root biomass were not affected by inter-specific competition nor inoculum source (*F*_1,50_ = 1.6, *P*>0.2; *F*_1,48_ = 0.6, *P*>0.4 for shoot and root biomass receptively, Table 2, Fig. 1b and d).

**Table 2:** Effects (ANOVA results) of inter-specific competition and soil source (soil microbial communities from continuously-grazed vs ungrazed grasslands) on shoot and root biomass of *Eriophorum vaginatum* and *Nardus stricta* plants growing in a glasshouse experiment.

Variation source	df	MS	*F* (*P*)
***Eriophorum vaginatum***			
**Shoot biomass**			
Inter-specific competition (C)	1	31.2	50.0 (<0.001)
Inoculum Source (S)	1	0.04	0.06 (0.80)
C×S	1	0.16	0.26 (0.60)
Error	47	0.62	
**Root biomass**			
C	1	2.07	26.0 (<0.001)
S	1	0.03	0.4 (0.52)
C×S	1	0.34	4.3 (.04)
Error	43	0.08	
***Nardus stricta***			
**Shoot biomass**			
C	1	<0.01	<0.01 (0.94)
S	1	0.02	1.6 (0.21)
C×S	1	<0.01	0.29 (0.60)
Error	50	0.01	
**Root biomass**			
C	1	0.05	0.91 (0.34)
S	1	0.04	0.64 (0.47)
C×S	1	<0.01	0.01 (0.90)
Error	48	.06	

df = freedom degrees. MS = mean squares, *F* = value of *F* statistic, *P* = associated probability of *F*.

### Plant N Uptake

**Figure 2:**
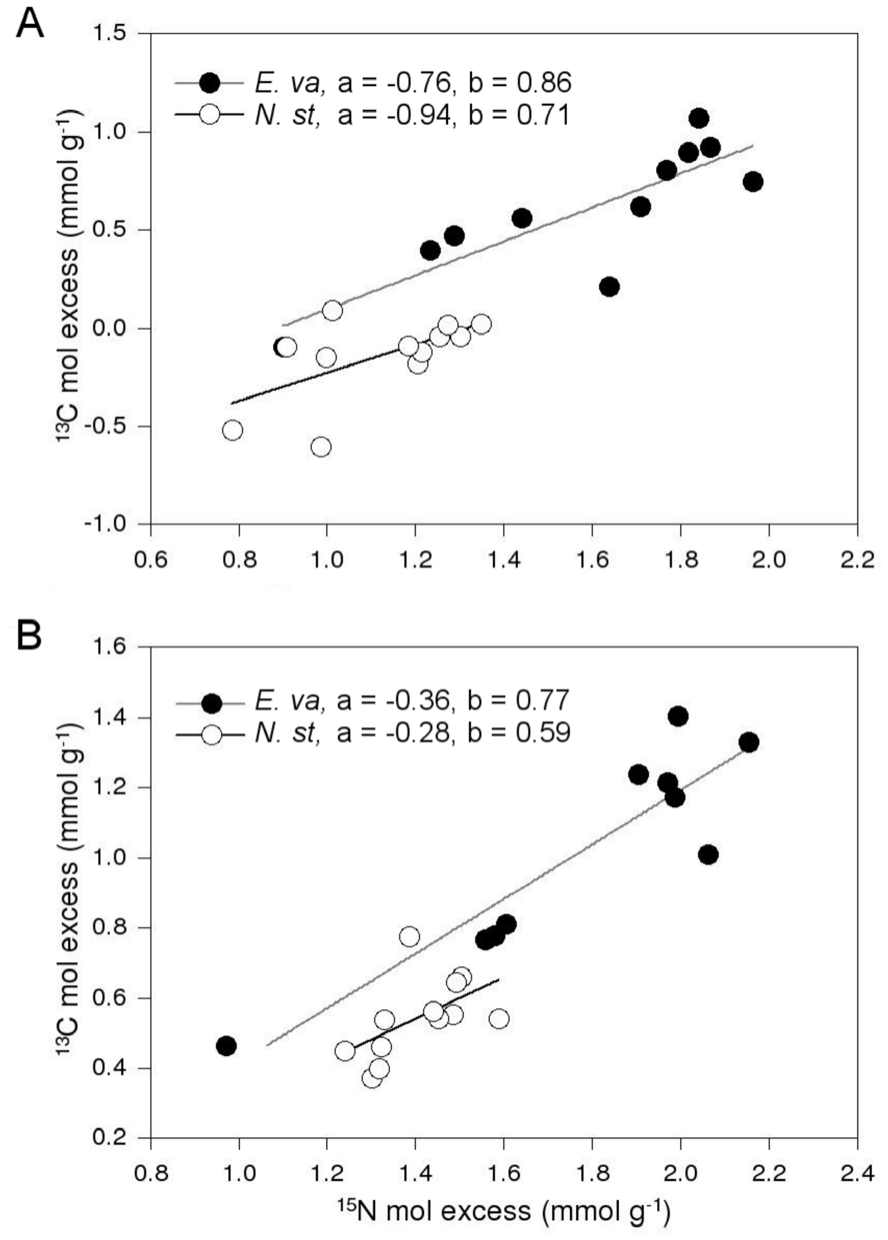
Isotopic enrichment in plant tissue. Regression analysis and parameter estimates (a = intercept with the ordinate, b = slope) of log transformed data of ^15^N against ^13^C mol excess for shoot (a) and root (b) biomass of plants treated with dual-labelled (^13^C and ^15^N) glycine+unlabelled NH_4_^+^. E. va = E. vaginatum, N. st = N. stricta.

**Figure 3:**
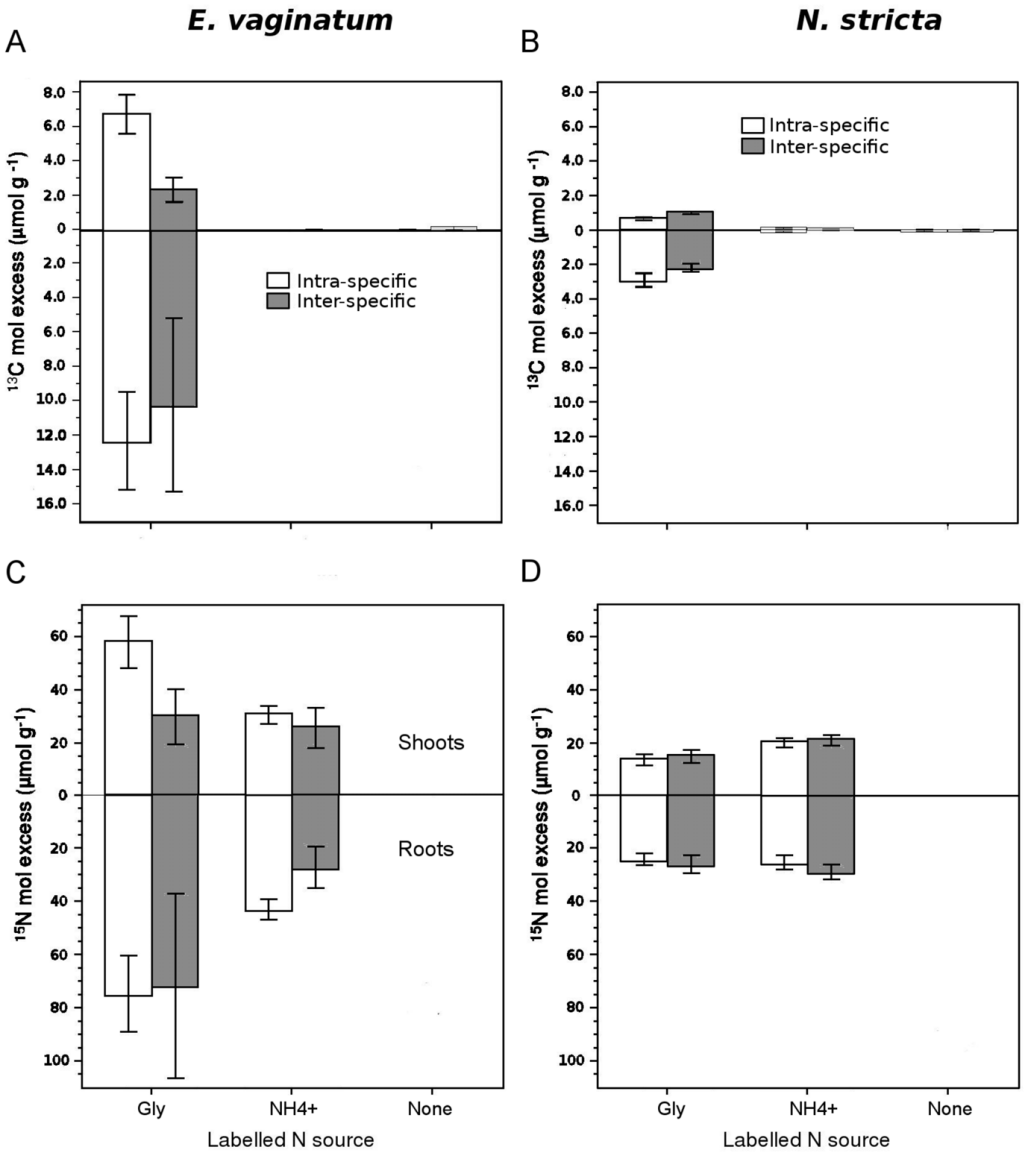
Plant competition and N sources uptake. Effects of competition on *E. vaginatum* and *N. stricta* ^13^C (a,b) and ^15^N (c,d) shoots (above the horizontal line on each plot) and roots (below the horizontal line on each plot) isotopic enrichment (expressed as molar excess) Data show plants grown experiencing intra- (C−) or inter-specific competition (C+) after applying N sources based on: glycine-ammonium solutions with isotopic dual-labelled (^13^C and ^15^N) glycine = Gly, ^15^N-labelled NH_4_^+^ = NH_4_^+^ or both compounds unlabelled = None. Values are means ±1 s.e. Note that whereas *E. vaginatum* molar excess is higher than that of. *N. stricta*, molar excess is expressed on a per plant biomass basis which was much lower for *E. vaginatum* (i.e., molar excess *per se* is not an indicator of competitive ability).

Linear regressions of log transformed data of ^13^C against ^15^N molar excess in the glycine labelled treatment showed that that both species likely took up intact glycine (*E. vaginatum* shoots: *F*_1,10_ = 19.0, *P*<0.01; roots: *F*_1,8_ = 38.5, *P*<0.001; *N. stricta* shoots: *F*_1,10_ = 5.5, *P*<0.05; roots: *F*_1,10_ = 4.0, *P* = 0.07; Fig. 2). There was no ^13^C or ^15^N enrichment in either species in the unlabelled negative control, indicating that experimental contamination of plant tissue was negligible (Fig. 3). Inoculum source did not have an effect on any of the molar excess values for roots or shoots in either plant species (not shown). A weakly significant species (within competition)×^15^N solution interaction for ^15^N molar excess in shoot biomass (*F*_2,34_ = 3.0, *P* = 0.06) provided evidence that our plant species displayed different N uptake preferences, and that plant species preferences were modified by inter-specific competition. Thus, *E. vaginatum* shoots’ ^15^N molar excess was 63% greater when N was supplied with labelled glycine than with labelled NH_4_^+^ (Fig. 3c). However, when experiencing inter-specific competition, *E. vaginatum* preference for glycine disappeared (Fig. 3c). On the other hand, *N. stricta* shoots’ ^15^N molar excess was 66% greater when supplied with labelled NH_4_^+^ than with labelled glycine (Fig. 3), and inter-specific competition did not affect this N source preference in this species. Inter-specific competition did not affect either ^13^C (*F*_1,15_ = 1.8, *P* = 0.19), or ^15^N enrichment in roots (*F*_1,37_ = 1.5, *P* = 0.22) for neither plant species (Fig. 3).

### Soil Microbial Properties

**Figure 4:**
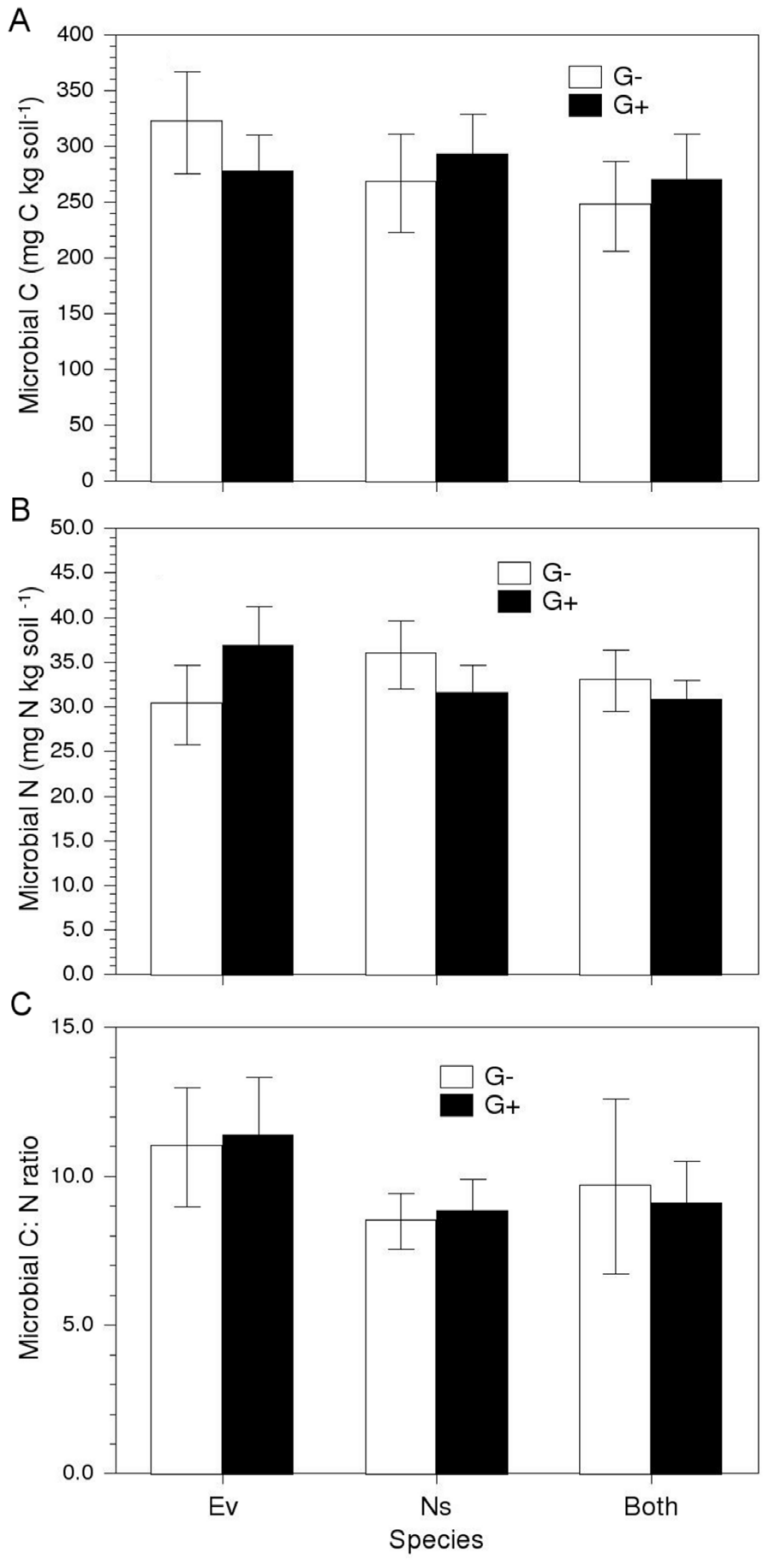
Soil properties and plant-soil feedbacks. Effects of microbial inoculum source (G+ vs G−) and pot-type (a proxy for plant species) on: (a) soil microbial biomass C; (b) soil microbial biomass N; and (c) microbial biomass C:N ratio. Soils were planted with *E. vaginatum* plants (Ev), or *N. stricta* plants (Ns) experiencing intra-specific competition only, or with both species under inter-specific competition (Both). Values are means ±1 s.e. Legends as in Fig. 1.

There were no differences between grazed and ungrazed inoculated soils in any of the measured microbial properties, namely microbial biomass C (*F*_1,70_ = 0.007, *P*>0.9), N (*F*_1,70_ = 0.06, *P*>0.7) and microbial biomass C:N ratio (*F*_1,66_ = 0.15, *P*>0.7) (Fig. 4). None of these variables responded to plant species identity (*F*_1,70_ = 0.15, *P*>0.8; *F*_1,70_ = 0.6, *P*>0.5; and *F*_1,66_ = 0.75, *P*>0.7, for soil microbial C, N and C:N ratio, respectively).

## Discussion

We investigated how functionally-distinctive soil microbial communities, as a result of long term differences in grazing management, and plant competition affected uptake of glycine (organic N = ON) and NH_4_^+^ (inorganic N = IN) by two coexisting graminoids, *E. vaginatum* and *N. stricta*. Abundances of these two graminoids in temperate acidic grasslands vary across grazing and soil moisture gradients, with *E. vaginatum* being of greater abundance under ungrazed and higher soil moisture conditions, and *N. stricta* being dominant in grazed grasslands [38]–[42]. Moreover, the ratio of ON to IN is typically higher in soils where *E. vaginatum* is more abundant [23]. Based on the ON to IN ratio, we predicted these two species would show different N uptake patterns, with *E. vaginatum* taking up more glycine than NH_4_^+^, and *N stricta* showing the opposite pattern. Consistent with this notion, we observed that *E. vaginatum* displayed greater uptake of glycine than *N. stricta,* even when both N chemical forms were added in similar concentrations. On the other hand, *N stricta* showed a higher uptake of NH_4_^+^. Preferential uptake of glycine over NH_4_^+^ by *E. vaginatum* has been shown elsewhere [43]. However, our observation that *N. stricta* took up more NH_4_^+^ than glycine contrasts with those of Weigelt et al. [7] who showed that *N. stricta* took up most N in the form of serine and glycine. Nevertheless, our results agree with those of other studies, which have not detected greater amino acid utilization over IN by this grass species [3], [9]. The greater capacity of *Nardus stricta* to uptake IN compared to *E. vaginatum*, as observed here, is consistent with results by Havill et al. [44], who found greater nitrate reductase activity after nitrate addition in *N. stricta* than in *E. vaginatum*. Previous studies have interpreted differentiation in uptake of N chemical forms as a mechanism contributing to local coexistence of plant species [2], [8], [45]. Unlike those studies, we interpret the differentiation in N chemical forms observed here as a result of habitat differentiation along gradients of grazing and soil properties in our two plant species. Thus, inter-specific differences in response to soil properties [39], [42], [46], and resistance to grazing [41], [47], [48], might have contributed to shape the differences in N uptake patterns between these two species.

We also hypothesized that functionally-distinctive soil microbial communities from grazed an ungrazed grassland would affect NH_4_^+^ and glycine uptake patterns by *E. vaginatum* and *N. stricta*. We based this hypothesis on past knowledge of how grazing influences the biomass, activity and structure of soil microbial communities, and how it enhances soil nutrient cycling [16], [19], [20], [49]. However, despite reported differences in microbial biomass N and activity of soil microbial communities in response to different grazing management in our two soil sources [21], [23], we found that different soil inoculums taken from grazed and ungrazed grassland did not affect plant N uptake of either plant species. Soil microbes grew after our soil inoculation, as evidenced by the soil microbial biomass C and N values at the beginning and at the end of the experiment. However, microbial biomass C was half that found in both the field [23] and in soil from a similar microcosm experiment [21]. Thus, the lack of effects of functionally-distinctive soil microbes on plant N uptake might have resulted from the small size of the microbial community in inoculated microcosms. Alternatively, this lack of effect might suggest that microbial communities of grazed and ungrazed grassland were functionally equivalent with respect to their effect of plant N uptake [50], [51]. This latter notion is broadly consistent with observations by Harrison et al. [9], who found no difference in uptake of ON and IN by soil microbial biomass of soils influenced by a range of plant species with contrasting life histories. However, Dunn et al. [15] directly manipulated soil microbial activity through the addition of glucose in soil and found that an increase in microbial activity altered patterns of ON and IN uptake in temperate grass species. Since we did not measure ^15^N enrichment in the microbial biomass, we are not able to distinguish between the two alternative interpretations, i.e. whether lack of effect was due to the microbial community small size or whether it reflects functional equivalence for plant uptake of different N forms between our microbial communities from grazed and ungrazed grasslands.

We also hypothesized that *N. stricta* would affect *E. vaginatum* N uptake more than it would be affected by *E. vaginatum* when both species competed. We based this hypothesis on the fact that *N. stricta* exhibits higher competitive ability traits (higher root biomass and capacity to reduce N availability in soil), and it has a greater negative impact on *E. vaginatum* performance than vice versa [21]. Mirroring the results on shoot and root biomass observed here and by Medina-Roldán et al. [21], *N. stricta* altered N uptake patterns of *E. vaginatum*, but it was not affected by inter-specific competition with *E. vaginatum*. The main effect of *N. stricta* inter-specific competition on *E. vaginatum* was a reduction in uptake of glycine by the latter, therefore increasing the proportional uptake of NH_4_^+^ by *E. vaginatum*. Since *N. stricta*’s main N source was NH_4_^+^, alteration of *E. vaginatum* N uptake patterns by competition with *N. stricta* is likely to have increased resource overlap in these two plant species. Ashton et al. [13] found that the superior competitor in an alpine grassland switched to different N forms (higher plasticity) when competing with other plant species, thus increasing resource complementarity. They hypothesized that this N-use plasticity could reduce resource niche overlap, therefore promoting plant coexistence in their alpine ecosystem. Unlike Ashton et al [13], we did not observe higher N-use plasticity in the superior competitor, but a switch in N-use by the inferior competitor that might have increased N niche overlap. Thus, a plant trait potentially related to higher competitive ability unexplored so far might be the capacity to modify preferences of IN and ON uptake in competitors, as *N. stricta* did on *E. vaginatum*. Increased niche overlap in N uptake patterns, and the findings on plant competition described in Medina-Roldán et al [21], suggest that the observed co-existence of *N. stricta* and *E. vaginatum* in a range of grazing and soil properties in semi-natural mountain habitats (Chadwick 1960; Wein 1973) is based on different mechanisms than those that reduce the intensity of competition. Such other mechanisms might rely on non-equilibrium dynamics caused by herbivores gap creation or heterogeneity in soil conditions prevalent in semi-natural mountain grasslands.

Finally, inter-specific competition with *N. stricta* reduced *E. vaginatum* performance overall, but this reduction was lower for *E. vaginatum* root mass on grazed in comparison to ungrazed soil. This plant-soil feedback result is in contrast to findings by Medina-Roldán et al. [21], where *E. vaginatum* root biomass negative response to inter-specific competition with *N. stricta* was larger on grazed than ungrazed soil. Since Medina-Roldán et al. [21] experiment was longer and included effects of plant density, we feel that their results are more reliable in the long-term for the implications of plant-soil feedbacks in the competition of these plant species.

In summary, we found that *E. vaginatum* and *N. stricta*, two dominant graminoids of temperate semi-natural-acid grassland differ in their patterns of NH_4_^+^ and glycine uptake, and that this might be related to the relative availabilities of ON and IN in the habitats where these species dominate. Specifically, we found that *E. vaginatum* takes up more glycine than NH_4_^+^, whereas the opposite is true for *N. stricta*. Furthermore, we found that inter-specific competition with *N. stricta* increased the proportional usage of NH_4_^+^ in *E. vaginatum*, the weaker competitor, increasing species resource overlap and likely intensity of competition. This last finding suggests that coexistence of these plant species in semi-natural mountain habitats is unlikely to be based on mechanisms that promote resource use complementarity. We hypothesize that coexistence in these species might rather be based on non-equilibrium mechanisms such as disturbance and gap creation caused by herbivores or heterogeneity in soil conditions.
